# A turning point in history: thinking about the unthinkable

**DOI:** 10.1007/s00411-022-00976-4

**Published:** 2022-04-10

**Authors:** Werner Rühm, Anna A. Friedl, Andrzej Wojcik

**Affiliations:** 1 Institute for Radiation Medicine, Helmholtz Center Munich, 85764 Neuherberg, Germany; 2 University Hospital, Ludwig-Maximilians-University LMU Munich, Munich, Germany; 3 MBW Department, Centre for Radiation Protection Research, Stockholm University, Stockholm, Sweden

Currently, we are watching—as probably most of our readers do—the abhorrent news that reach us from Ukraine, with numerous casualties among the civil population and millions of refugees who desperately try to find a safe place for themselves and their loved ones. This tragedy is just the beginning of a long and painful story, it will affect millions of innocent people and determine their future life for decades. On top of this comes the threat of a nuclear tragedy triggered by the invasion of Ukraine.

This journal—*Radiation and Environmental Biophysics (REBS)*—was founded in 1962, at a time of major crises during the Cold War. In the current portfolio of Springer Nature, which includes approximately 3,000 journals, REBS is one of the very few that focus on the biological effects of ionizing radiation with a major emphasis on health effects in humans. By running this journal we, as all editors of REBS did before, support the scientific community in their efforts to investigate radiation effects towards a safe use of radiation technologies for the global benefit of all humans.

The invasion of Ukraine is unacceptable, and it may have some long-term implications for the topics covered by REBS. The occupation of the Chornobyl exclusion zone (where the shelter of the damaged unit 4 and three other closed units of the Chornobyl reactor are located) by Russian troops has a symbolic meaning given that, globally, “Chornobyl” is associated with the largest nuclear accident to date. As such already unthinkable, the military actions in Ukraine escalated further and we had to follow news that shells had been fired towards a running nuclear power plant (NPP), the Zaporizhzhia facility in southern Ukraine. This raised fears that an accident like the one in Fukushima, Japan, where a nuclear meltdown led to a release of high amounts of radioactivity into the environment, could become real. Although in Japan this event was triggered by a tsunami, the evident question is whether for the first time in human history an active military operation in a country with running NPPs could cause a nuclear disaster. And even worse, some statements of politicians seemed to indicate that use of nuclear weapons is not completely ruled out. It is no surprise that people all over the world are concerned. Some even consider preparing themselves and their families against a nuclear detonation. To make a long story short—within the past few days the unthinkable has become thinkable, and the thinkable has already, at least in part, become reality!

So, is there anything we as scientists working in the field of radiation research and radiation protection could do to meet these concerns of the worldwide public? Yes—be prepared to serve with expert judgement and advise to the public on detrimental health effects of exposure to ionizing radiation and ways of their mitigation.

Radiation effects are complex, and the public opinion is easily misled by prejudiced opinions and rumors exaggerating or trivializing health effects. This contributes to anxiety and destabilization of the much needed social order. However, there is already sound scientific understanding of radiation effects in general. It is important that we as scientists are well familiar with validated and reliable knowledge about health consequences of acute and prolonged radiation exposure. Reliable sources of knowledge are peer-reviewed journals publishing articles with reproducible results, and review documents published by international organizations such as the United Nations Scientific Committee on the Effects of Atomic Radiation (UNSCEAR) (www.unscear.org) and the International Commission on Radiological Protection (ICRP) (www.icrp.org). It may be helpful to have at hand and become familiar with reports on the consequences of the Chornobyl and Fukushima-Daiichi NPP disasters, such as (UNSCEAR 2008, 2020/2021; ICRP 2020, 2021).

Still, many knowledge gaps remain and require further research, especially concerning scenarios hitherto regarded as unthinkable. It is a regrettable and sad sign of time that we have now to start thinking about them.
